# First Molecular Identification and Clinical Presentation of Crenosomosis in a Dog from Slovakia

**DOI:** 10.1007/s11686-024-00861-8

**Published:** 2024-06-29

**Authors:** Michaela Kaduková, Martin Kožár, Andrea Schreiberová, Barbora Šišková, Gabriela Štrkolcová

**Affiliations:** 1 Department of Epizootiology, Parasitology and Protection of One Health, University of Veterinary Medicine and Pharmacy in Košice, Komenského 73, 041 01 Kosice, Slovakia; 2 Small Animal Clinic, The University of Veterinary Medicine and Pharmacy in Kosice, Komenského 73, 040 01 Kosice, Slovakia

**Keywords:** *Crenosoma vulpis*, Bronchoscopy, Dog, Foxes, Molecular identification, Baermann technique

## Abstract

**Purpose:**

*Crenosoma vulpis* (Dujardin,1845) is a lungworm which has spread worldwide in canines and is associated with upper respiratory infections. In a majority of cases, the infections are accompanied with chronic cough. Diagnosis of lungworms is often underdiagnosed and can be misinterpreted as other respiratory diseases.

**Methods:**

The Small Animal Clinic of the University Veterinary Hospital admitted an 11-month-old dog presented with persistent cough associated with difficulty in breathing and even asphyxia. Based on clinical symptoms, the patient underwent radiological and bronchoscopic examination. Bronchoscopy revealed the presence of lungworms obturating the branches of the tracheobronchial tree. Larvae were collected by bronchoscopic lavage and subjected to parasitological and molecular examination.

**Results:**

Microscopic detection and morphological identification of the worms removed during the bronchoscopy confirmed the presence of female adult worms. The subsequent molecular characterisation of the mitochondrial (cytochrome c oxidase subunit I gene (cox1) and 12S ribosomal DNA (rDNA)), nuclear (18S rDNA) genes, as well as the analysis of the second internal transcribed spacer (ITS-2) region of the ribosomal DNA, confirmed the *Crenosoma vulpis* species*.* Faecal samples were processed using the Baermann method, which confirmed the presence of the larval stage 1 of *C.* *vulpis*. The therapy with fenbendazole at a dose of 50 mg/kg of live weight once daily for the period of 7 days was initiated for the patient.

**Conclusion:**

This paper presents the first molecularly confirmed clinical case of a *Crenosoma vulpis* infection in an 11-month-old female dog of the Miniature Schnauzer breed in Slovakia.

## Introduction

*Crenosoma vulpi*s (Nematoda: Metastrongyloidea), also known as the fox lungworm, is a member of the Crenosomatidae family and it is primarily associated with respiratory infections in canines. It mainly occurs in red foxes (*Vulpes vulpes*), less frequently in dogs (*Canis lupus familiaris*), arctic foxes (*Alopex lagopus*), gray foxes (*Urocyon cinereoargentaus*), wolves (*Canis lupus*), coyotes (*Canis latrans*), and European badgers (*Meles meles*); it is also present in European pine martens (*Martes martes*) and beech martens (*Martes foina*), while from the endemic point of view, it usually lives in the Northern America and Europe [4, 16, 30]. As a result of free movement of foxes in the wild nature without having any natural animal predator, and due to the fact that they stay near the residential areas and tourist locations, foxes are considered to be the main cause of the spread of lungworms to non-endemic regions and as the source of this infection for carnivores [18, 38]. *C. vulpis* was also reported in foxes in Africa and Algeria [35].

Stunženas and Binkiene (2021) [47] stated in their study that the *Crenosoma* genus includes 14 validated species, while the most widespread species in Europe is the *C. vulpis* fox lungworm. A rather frequently occurring *Crenosoma striatum* species was confirmed mainly in European hedgehogs (*Erinaceus europeus*) in Portugal and Italy [31]. Other species detected in Europe include *Crenosoma melesi*–primarily in European badgers (*Meles meles*), as well as *Crenosoma petrowi*–identified in European badgers (*Meles meles*), European pine martens (*Martes martes*) and beech martens (*Martes foina*) in Romania [16].

The life cycle of *Crenosoma vulpis* is indirect. The intermediate hosts include land slugs, such as *Arion vulgaris* and *Limax maximus,* as well as common garden snails *Cornu aspersum* [9, 21]. A definitive host acquires the infection by ingesting an intermediate host whose tissues contain a developing infectious L3 larvae. After the definitive host ingests the infectious larval stage, the larvae migrate through the gastrointestinal tract and via the lymph, and through their subsequent migration through the liver, heart and lungs they reach the locations in the lungs where they transform into sexually mature adults. Females are ovoviviparous; L1 larvae are released from the eggs and after a short period of time they are coughed up and swallowed so they get to the intestine and are eventually released to the external environment with faeces. Adult parasites may live in a definite host as long as 10 months, and their prepatent period is 18–21 days [18, 21, 44]. Depending on the parasite load, clinical symptoms may vary from the asymptomatic form to the nasal discharge, dyspnoea and chronic coughing, which is caused by the irritation of the lungs and bronchi by the parasite [11].

The objective of this paper was to identify the lungworm in a dog with clinical manifestations by applying morphology-molecular analyses.

## Material and Methods

### Case Presentation/Investigation

In the article, a clinical case of a patient that was referred to the clinic below due to several weeks persisting cough associated with difficulty in breathing and even suffocation is described. The patient–a female dog of the Miniature Schnauzer breed, with the weight of 6.4 kg and the age of 11 months, was treated at a private veterinary clinic, where he was administered two groups of antibiotic therapy and the medication from the group of glucocorticoids. Due to the failure of the conventional therapy, the patient was referred to the Small Animal Clinic of the University Veterinary Hospital at the University of Veterinary Medicine and Pharmacy in Košice, for an endoscopy. The clinical examination did not reveal any significant pathological changes in the patient’s condition (CRT = 2 s; N =  ≤ 2 s), light-pink mucous membranes, in the shock stage, with present normothermia (T = 38.3 °C), the animal was slightly excited, its breathing was shallow and of the costo-abdominal type.

## Radiography

**Fig. 1:**
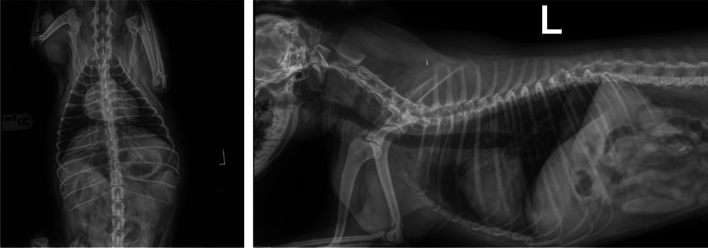
Radiography

Subsequently, an X-ray examination of the chest cavity was performed on the LL and VD projection with the finding of a change in the trachea which was dorsally shifted away, the bronchial lung pattern, significant sharping of the lung field, and a change in the bifurcation region and the heart region (Fig. 1). The summary of all clinical signs indicated.

the need for an additional specific diagnostic examination by a direct visualisation of the lower respiratory tract.

## Bronchoscopy

The intravenous access was applied (*v. cephalica*) and venous blood was collected for haematological and biochemical analyses, with the finding of a slight decline in the eosinophil and reticulocyte counts. The patient was then sedated with butorphanol (Butomidor, RP Richter Pharma, Austria) at a dose of 0.2 mg/kg of live weight and medetomidine (Cepetor, CP-Pharma, Germany) at a dose of 0.02 mg/kg of live weight. Subsequently, the patient was put under general anaesthesia with an injection of diazepam (Apaurin, Krka d.d., Slovenia) at a dose of 0.2 mg/kg of live weight and propofol (Propofol, Fresenius Kabi) at a dose of 3 mg/kg of live weight. The patient was maintained under general anaesthesia with hyperoxygenation of the lower respiratory tract.

**Fig. 2:**
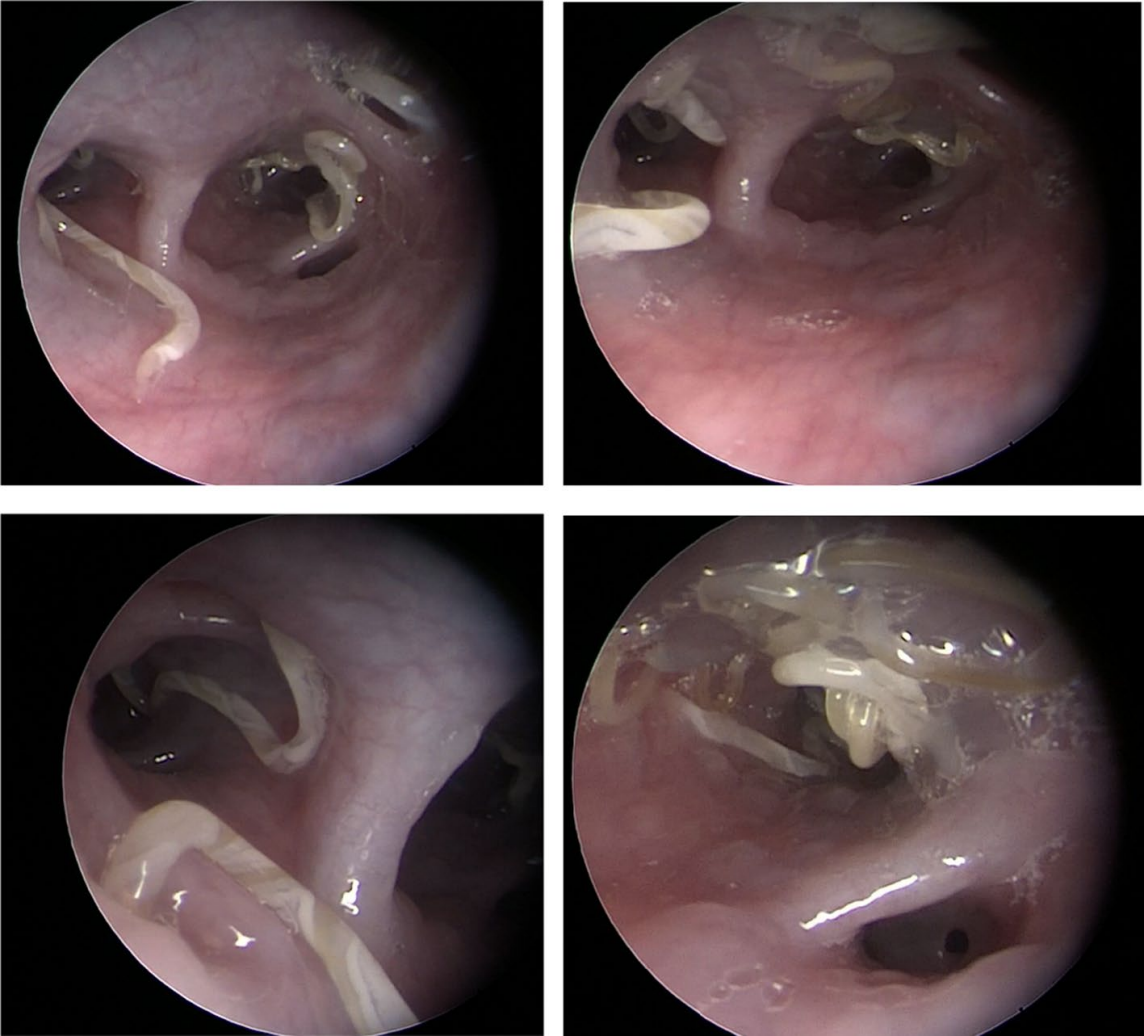
Bronchoscopy

Following the sedation of the patient, endoscopy of the oral cavity was performed, with the finding of a change in the tonsil region, in particular enlarged tonsils with chronic inflammation. In the rima glottidis region, slight hyperaemia was observed, as well as the finding of prominent polyp formations on the cartilage base of the rima glottidis. After reaching the larynx region, significant swelling and hyperaemia of the mucous membrane was observed in the entire trachea, while the vessels were significantly hyperaemic as well. The bifurcation region showed a typical image of chronic irritation of the lower respiratory tract with hyperaemia and dorsal suppression of the trachea to 30%. Near the branches of the bronchi and the bronchioles, a chronic condition of the mucous membrane was observed, corresponding to the hyperplastic changes in the epithelium with a large amount of foamy exudate. After reaching the caudal regions and exhausting the effusion, the dominant finding was the presence of lungworms obturating the branches of the tracheobronchial tree (Fig. 2). The nostril region did not contain any effusion or depigmentation. After reaching the nasal cavities, hyperaemic changes in the mucosa were observed, as well as the hyperaemic vessels and a swelling with a small amount of viscous phlegm.

## Morphological and Molecular Identification of Lungworms

The lungworms collected during the bronchoscopy and the patient’s faeces were subsequently examined at the Department of Epizootiology, Parasitology and Protection of One Health. They were subjected to the microscopic and coprological diagnostics, followed by the molecular identification. All microscopic images and measurements were made using the light microscopy in the PROMICRA Introduces QuickPHOTO 3.0 Microscopy Imaging Software. The faeces of the treated dog, as well as the faeces of the dogs that lived in the same household, were examined by applying the coprological Baermann technique.

Genomic DNA was extracted from 4 adult female lungworms using a commercial kit (DNeasy Blood & Tissue Kit, Qiagen, GmbH, Hilden, Germany) following the manufacturer’s instructions. The molecular identification of the lungworms was carried out by amplification through a polymerase chain reaction (PCR) of 4 different DNA regions – genes of the mitochondrial DNA (cox1 and 12S rDNA) and nuclear DNA (18S rDNA), and based on the analysis of the ITS-2 region of rDNA. The extracted DNA was used as a template for the PCR amplification of an approximately 710 bp region of the mitochondrial cox1 gene with a pair of “universal” primers, widely used for the invertebrate species: LCO1490 forward primer (5’-GGTCAACAAATCATAAAGATATTGG-3′) and HCO2198 reverse primer (5’-TAAACTTCAGGGTGACCAAAAAATCA-3′) [16, 20, 45] Partial fragments of mitochondrial 12S rRNA (330 bp) and nuclear 18S rRNA (1700 bp) genes were amplified by the conventional PCR using two sets of primers (12SF forward primer: 5′-CGGGAGTAAAGTTTTGTTTAAACCG-3’ and 12SR reverse primer: 5′-CATTGACGGATGGTTTGTACCAC-3′) and (NC18SF1 forward primer: 5′-AAAGATTAAGCCATGCA-3′ and NC5BR reverse primer: 5′-GCAGGTTCACCTACAGAT-3′, respectively) designed by Latrofa et al. 2015. The more variable ITS regions of the ribosomal RNA genes were used to amplify the second internal transcribed spacer ribosomal DNA sequences using the universal direct primers for nematodes (NC16 forward primer: 5′-AGTTCAATCGCAATGGCTT-3′ and NC2 reverse primer: 5′-TTAGTTTCTTTTCCTCCGCT-3′) of 1,250 bp in size [27].

All resulting PCR products were sent to the Microsynth Seqlab (Vienna, Austria) or SEQme (Dobříš, Czech Republic) for purification and sequencing in both strands with the identical primers used for the PCR. The sequencing was performed by the Sanger sequencing method. Resulting sequences were analysed and edited using MEGA X software [29]. and the assemblage of the nucleotide sequences was carried out in Gene Tool Lite 1.0 software (BioTools Inc., Jupiter, FL, USA). The consensus sequences were compared with the sequences deposited in GenBank by applying the nucleotide BLAST algorithm (https://blast.ncbi.nlm.nih.gov/Blast.cgi). The sequences from this study for the cox1, 12S, 18S genes and the ITS-2 region were deposited in GenBank under unique accession numbers (Table 1). For the purpose of a phylogenetic analysis of the cox1 gene, all the sequences of *Crenosoma* spp. available in the GenBank were selected. The sequences were aligned and the phylogenetic tree of the gene was constructed using the MEGA X software [29]. The phylogenetic analysis was inferred using the statistical method of the Neighbour-Joining algorithm. The optimal tree is shown, with the sum of the branch length of 0.35541990. The percentages of the replicate trees, in which the associated taxa clustered together in the bootstrap test (1,000 replicates), are shown next to the branches. The tree was drawn to a scale, with the branch lengths in the same units as those of the evolutionary distances used to infer the phylogenetic tree. The evolutionary distances were computed using the Maximum Composite Likelihood method, and they are in the units of the number of base substitutions per site. All positions containing gaps and missing data were eliminated (complete deletion option). The analysis involved 29 nucleotide sequences. There were a total of 441 positions in the final dataset (Fig. 5).

**Table 1:** Accession unique numbers for the sequences *C. vulpis*: 12S rRNA, 18S rRNA, ITS2 region and cox1 gene deposited in the GenBank. (n/d not detected)

Isolates	12S rRNA gene	18S rRNA gene	ITS2 region	cox1 gene
Cr1Dog1	PP109372 (haplotype I)	n/d	PP106991	PP106438
Cr2Dog1	PP109373 (haplotype II)	PP107880	PP106992	PP106439
Cr3Dog1	n/d	PP107881	PP106993	PP106440
Cr4Dog1	n/d	PP107882	PP106994	PP106441

## Results

### Morphological and Coprological Diagnostic

**Fig. 3:**
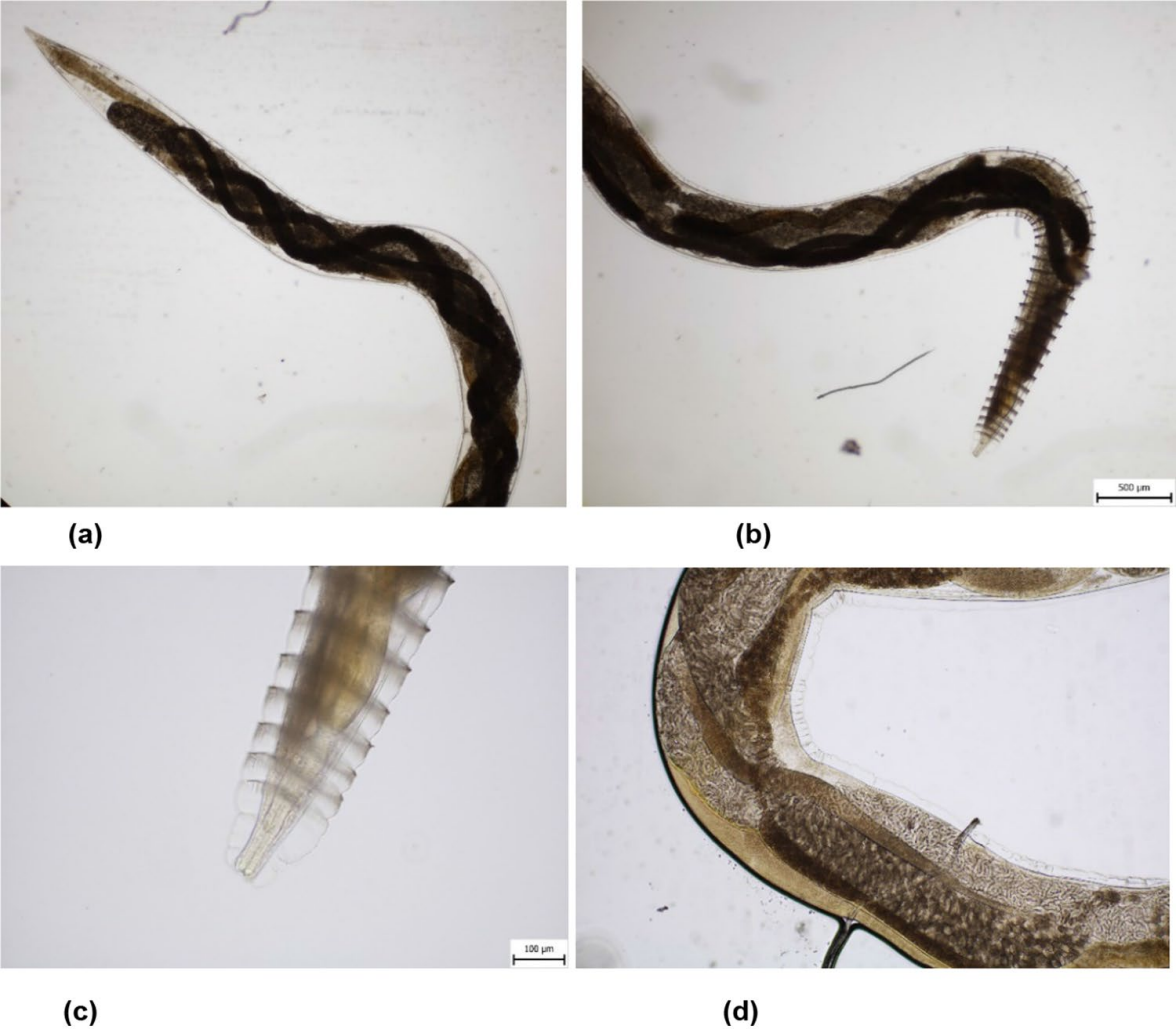
Morphological diagnosis of *Crenosoma vulpis*. **a** (posterior part of *C. vulpis*), **b** (anterior part of *C. vulpis*), **c** (front part with ring-shaped folds of the cuticle and numerous thorns), **d** (female of *C. vulpis* with the uterus filled with the first-stage of larvae)

The microscopic diagnostics of the worms collected during bronchoscopy revealed the presence of adult females bearing multiple larvae in their wombs. Based on the morphological features of the parasite (the presence of typical cuticular ridges on the front end; in females, stretched cuticular folds on the posterior end; a visible anus) (Fig. 3), *C. vulpis* was identified [16].

**Fig. 4:**
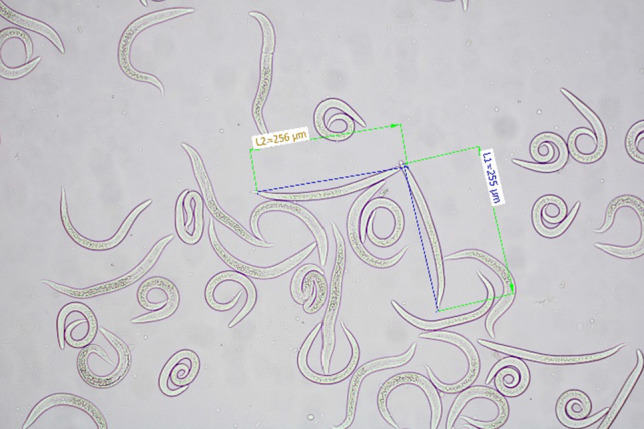
Baermann technique–first stage of larvae

The faeces of the dog were examined by the coprological Baermann technique, and the presence of stage 1 larvae (L1) was revealed. The size of the larvae ranged from 270 to 290 μm (Fig. 4). The average length of the L1 larvae ranges from 243 μm to 281 μm [12, 34]. In addition, faecal samples of four dogs that lived in the same household were examined by applying the Baermann method; however, the presence of *C.* *vulpis* larvae was not confirmed.

## Molecular and Phylogenetic Analysis

The molecular characterisation of the mitochondrial cox1 and 12S rDNA genes and nuclear 18S rDNA genes, and based on the analysis of the ITS-2 region of rDNA, the presence of the *Crenosoma vulpis* species was confirmed. For the 12S ribosomal RNA gene, 2 high-quality sequences were obtained and compared using the BLAST tool in GenBank; they were a 100% match to the *C. vulpis* KR920039 sequence (haplotype I; hosts: *Vulpes vulpes*, *Canis lupus familiaris, Meles meles*) and the KR920040 sequence (haplotype I; host: *Vulpes vulpes)*, both originated in Italy (Latrofa et al. 2015). Based on that comparison, two haplotypes were determined – for the Cr1Dog1 sample (PP109372) as haplotype I, and for the Cr2Dog1 sample (PP109373) as haplotype II (Table 1). Our sequences for the 18S rRNA gene, deposited under numbers PP107880–PP107882, were compared in GenBank to the *C. vulpis* KR920038 sequence (hosts: *Vulpes vulpes, Canis lupus familiaris, Meles meles* from Italy; Latrofa et al., 2015) [30]; and the AJ920367 sequence (host: *Vulpes vulpe*s from Canada; Chilton et al., 2006) [26]; with the identity ranging from 99.60% to 99.73%. Sequences (PP106991–PP106994) for the ITS2 region were compared to OM480716 (host: *Canis lupus familiaris* from USA; Pohly et al. 2022) [40]; MT808324–MT808325 (host: *Vulpes vulpes* from United Kingdom; Allen et al. 2020) with the 100% identity, and compared to KF836608 (host: *Vulpes vulpes* from Germany; Schug et al. 2018) [46] with the identity of 99.41%–99.75%. In the analysis of the cox1 mtDNA gene, four our sequences (PP106438–PP106441) exhibited the 98.10%–99.58% nucleotide identity to the KM216824 sequence of *C. vulpis* deposited in GenBank (host: *Vulpes vulpes* from Germany; Schug et al., 2018) [46].

**Fig. 5:**
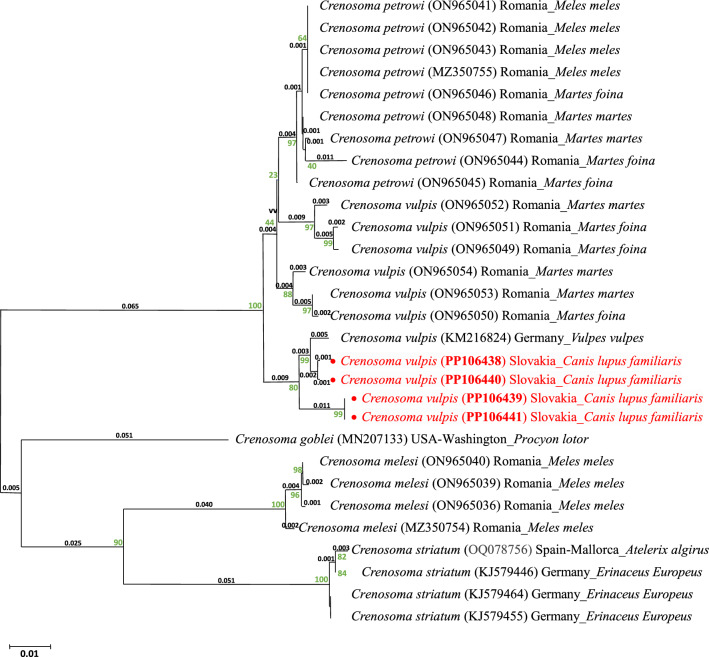
The tree were constructed using the Neighbour-Joining method (NJ) and depicting the relationships among *Crenosoma vulpis, Crenosoma petrowi, Crenosoma goblei, Crenosoma melesi* and *Crenosoma striatum* based on gene cox1 mtDNA sequences data available in theGenBank database

To investigate the phylogenetic relationships among the species of the *Crenosoma* genus, we selected the PP106438–PP106441 sequences for the cox1 gene of *C. vulpes* from this study (host: *Canis lupus familiaris* from Slovakia), as well as all the available reference sequences from GenBank at NCBI for *C. vulpes, C. petrowi, C. melesi, C. striatum*, and *C. goblei* from a variety of hosts and different geographic regions. The following sequences of cox1 gene were phylogenetically analysed: *C. vulpes* KM216824 (host: *Vulpes vulpes* from Germany; Schug et al. 2018) [46]; ON965049–ON965051 (host: *Martes foina* from Romania); ON965052–ON965054 (host: *Martes martes* from Romania) (Latrofa et al. 2015); *C. petrowi* MZ350755, ON965041–ON965043 (host: *Meles meles* from Romania); ON965047 and ON965048 (host: *Martes martes* from Romania); ON965044–ON965046 (host: *Martes foina* from Romania) (Latrofa et al. 2015); *C. melesi* ON965036, ON965039, ON965040 and MZ350754 (host: *Meles meles* from Romania) (Latrofa et al. 2015); *C. striatum* OQ078756 (host: *Atelerix algirus* from Mallorca, Spain); KJ579446, KJ579455 and KJ579464 (host: *Erinaceus Europeus* from Germany); and *C. goblei* MN207133 (host: *Procyon lotor* from Washington, USA) [23]. The phylogenetic tree revealed that *C. vulpis* and *C. petrowi* constituted a shared clade, even though they divided within the clad into further branches. A majority of the *C. petrowi* sequences exhibited high homology with a very small number of nucleotide changes; as for *C. vulpis,* there was a nucleotide variability among the sequences so they split into several smaller branches. *C. striatum*, *C. melesi* and *C. goblei* formed three separate related branches in the phylogenetic tree (Fig. 5).

## Treatment of *Crenosoma vulpis*

Based on the findings, the therapy with fenbendazole (Helmigal, PHARMAGAL spol. s r.o., Slovak Republic) at a, oral dose of 50 mg/kg of live weight once daily for the period of 7 days was indicated for the patient, concurrently with the administration of adjuvant preparations for the regeneration of mucous membranes – vitamin A, vitamin D3 (Aquavit ad3, PHARMAGAL spol. s r.o., Slovak Republic) at a dose of 1.5 ml pro toto once daily for the period of 2 weeks; the dosage regimen was q24h during the first week and q48h during the following 7 days.

Two weeks after the completion of the targeted therapy, the coprological examination was repeated by applying the Baermann method. The finding was negative and the X-ray scan of the lung region exhibited a significant improvement compared to the primary condition of the patient.

## Discussion

The present study discloses the first clinical case of crenosomosis with the morphological diagnostics and molecular characterisation of the *Crenosoma vulpis* species in a domestic dog (*Canis lupus familiaris*) in Slovakia. In a study by Čabanová et al. (2018a) [14], *C. vulpis* was confirmed by the Baerman method in a dog for the first time in Slovakia, while the presence of *C.* *vulpis* in red foxes was detected as early as in 1960s and 1980s in the research conducted by Mituch (1962) [36].

Crenosomosis induced by *Crenosoma* spp. nematodes may play an important role in chronic respiratory diseases of dogs in Europe [10]. The first such case was reported from the United Kingdom [8]. Since then, the cases of crenosomosis in dogs have been reported from several European countries, such as Ireland, Switzerland, Germany, Italy, Denmark, Belgium, Spain, Austria, Lithuania, France and the Czech Republic [2, 3, 5, 7, 21, 25, 32, 41, 43, 50, 51]. In a majority of those cases, productive cough developed into the chronic form, and dribbled saliva and breathlessness were present. Bronchoscopy revealed the hyperaemic trachea or the presence of mucopurulent exudate.

In the confirmed case of crenosomosis in a dog described herein, the animal was treated with fenbendazole at a dose of 50 mg/kg of live weight once daily per os for the period of 7 days. While the study by Caron et al. (2014) [7] claims that the 7-day therapy with fenbendazole could not cure the infection, and that a better effect would be achieved by a one-time local application of 10% imidacloprid combined with 2.5% moxidectin at a dose of 0.1 ml/kg of live weight, no efficient anthelminthic drugs specific for this parasite are currently marketed. With the use of febantel, fenbendazole, ivermectin and milbemycin oxime, a successful result of the therapy with the absolutely disappeared clinical signs and without the presence of L1 larvae in faeces was confirmed in several studies [4, 8, 39]. In the study by Conboy et al. (2013) [13] with dogs that were experimentally infected with *Crenosoma vulpis,* the therapy with milbemycin oxime (0.5 mg/kg) and praziquantel (5 mg/kg) was applied with a 98.7% efficiency.

In the neighbouring Czech Republic, Husník et al. (2011) [25] confirmed *C. vulpis* in a 1-year-old female dog of the Shetland Sheepdog breed. The patient was presented with tachypnoea and moist cough, and bronchoscopy revealed the hyperaemic trachea and phlegm-purulent exudate. Adults were collected with the use of bronchoalveolar lavage and after the microscopic analysis, the larvae were identified as *Crenosoma vulpis*. The application of the Baermann sedimentation method confirmed the presence of stage 1 larvae (L1). Similarly to the present study, the therapy indicated for the patient included fenbendazole (50 mg/kg) once daily for the period of 3 days, but it was combined with doxycycline (5 mg/kg) per os twice daily for the period of two weeks. That therapy too has been proven efficient.

At present, there is no evidence that gender or age represent a potential predisposition to this parasitic disease. It is assumed that dogs usually acquire the infection at the age of approximately 1 year [4, 33]. This assumption was confirmed by the present study, since the infection was detected in an 11-year-old dog. One of the risk factors that affect the outbreak of the disease is the living environment. The dogs that live in rural regions are exposed to a higher risk of infection than the dogs in urban regions due to the potential presence of foxes and a higher concentration of intermediate hosts [48].

Foxes as the most frequent reservoirs of cardiopulmonary parasites, including *C. vulpis*, are responsible for the extension of the geographical distribution of *C. vulpis* to urban regions in Europe [18, 30, 49]. Čabanová et al. (2018b) [13] confirmed *C. vulpis* in foxes in 17.51% of cases, and according to the existing data, foxes inhabit as much as 93.5% of the territory in Slovakia [28]. Out of all European countries, the highest prevalence of crenosomosis was reported from Norway 58.2%; Lithuania 53.8%; Bosnia and Herzegovina 45.3%; Pyrenees 44.8%; Portugal 39.29%; Germany 32.6%; Romania 32.0% and Italy 28.4% [6, 15, 17, 19, 22, 24, 37, 46].

In Europe, Latrofa et al. (2015) was the first team to confirm by a molecular analysis the presence of *C.* *vulpis* in dogs in Italy; later in 2022, it was confirmed by Remesar et al. in Spain. At present, there is only a very little available data in GenBank about the *C. vulpis* species, not only with regard to dogs as hosts, but also general data about the species. Molecular detection based on the database confirmed *C. vulpis* in the European countries and in the Northern America, while the most frequent definite hosts were *Vulpes vulpes* (Italy, Canada, United Kingdom, Germany, Bosnia and Herzegovina); *Canis lupus familiaris* (Italy, Spain, USA); *Meles meles* (Italy, Romania); and *Martes foina* and *Martes martes* (Romania) [16, 24, 26, 30, 40, 42, 46].

Species identification was carried out using the "universal" DNA primers – LCO 1490 and HCO 2198, which were originally intended for the amplification of highly-conservated regions of mitochondrial genes cytochrome c oxidase subunit I (cox1) in several taxons of invertebrates [16, 20]. The phylogenetic tree of the cox1 gene was compiled out of all sequences of *Crenosoma* spp. available in GenBank. The phylogenetic analysis for this gene showed a nucleotide variability among the sequences obtained from *C. vulpis,* and divided them into several smaller branches. One branch represents our sequences from Slovakia, which together with the sequences from Germany (host: *Vulpes vulpes*) constitute a homologue group, unlike other sequences obtained from Romania. It is assumed that this variability may be associated with the geographical spread of this parasite and with the diversity of its hosts. The *C. vulpis* and *C. petrowi* species are morphologically and genetically related. A comparison of sequences of the cox1 gene of those two species in GenBank revealed a high degree of percentual identity; moreover, a phylogenetic analysis showed a high degree of the relationship between *C. petrowi* and *C. vulpis* [16]. Since the GenBank does not contain any sequences of 12S, 18S or ITS-2 for *C. petrowi,* it is impossible to subject them to a phylogenetic analysis together with our sequences for *C. vulpis*. However, there is currently only a very little available data on the occurrence of *C. petrowi* in canines, since it was mostly detected in Eurasia and America (Addison et al. 1994) [1, 16]. Since the two species exhibited a high degree of relationship, *C. vulpis* was confirmed in our study by using also other genes (12S and 18S genes, ITS-2 region,). In the study by Latrofa et al. (2015) [30] conducted in Italy with *Vulpes vulpes, Canis lupus familiaris* and *Meles meles,* four haplotypes (I–IV) were identified based on the 12S rRNA target gene for *C. vulpis*, while our two *C. vulpis* lungworms were categorised as haplotypes I and II. An interesting fact is that both haplotypes I and II had the same host, in our case a dog, whereas in the aforementioned Italian study, as much as 3 haplotypes were detected in a single *Vulpes vulpes* individual*.* According to Latrofa et al. (2015) [30] haplotype I ranks among the most frequently occurring haplotypes in various hosts in Romania. Hodžić et al. (2016) [24] confirmed a new haplotype V for *C. vulpis* in the *Vulpes vulpes* foxes population in Bosnia and Herzegovina.

The most frequently used detection method is the Baermann technique, which is regarded as the most efficient method for the diagnostics of *C. vulpis.* It is cost-effective and easy to perform, but it is rarely used in veterinary clinics [48]. Rinaldi et al. (2007) [43] performed the detection of *C. vulpis* by applying the FLOTAC technique, and compared their results with the standard copro-microscopic methods: Baermann technique, McMaster technique, faecal flotation, and the Wisconsin method. The results showed that the FLOTAC method confirmed a larger number of larvae per gram of faeces when compared to the other methods. The findings obtained in the study indicate a potential improvement in the exact diagnostics of the lungworm infection in dogs.

## Conclusion

An increase in the population of foxes in Europe, as well as their more and more frequent migration across the urbanised regions, may result in the elevated numbers of infections in domestic dogs. Infection caused by the *Crenosoma vulpis* species in domestic dogs is generally regarded as rare, but it may often be overlooked. The spread of this parasite across Slovakia or even Europe may therefore be actually much more extensive than currently assumed. In cases where persisting cough and lung lesions are present, especially in young dogs, veterinary doctors should consider a potential presence of *C. vulpis,* as well as other lungworms, and include the Baermann technique in the routine examination methods intended for lungworms.

For the first time, the clinical presence of *C. vulpis* is confirmed in dogs in Slovakia via molecular analyses.

## Data Availability

The data supporting the findings of this study are available within the article.
